# Post-cardiotomy ECMO in pediatric and congenital heart surgery: impact of team training and equipment in the results

**DOI:** 10.5935/1678-9741.20150053

**Published:** 2015

**Authors:** Leonardo Augusto Miana, Luiz Fernando Canêo, Carla Tanamati, Juliano Gomes Penha, Vanessa Alves Guimarães, Nana Miura, Filomena Regina Barbosa Gomes Galas, Marcelo Biscegli Jatene

**Affiliations:** 1 Heart Institute of the Clinics Hospital of the Medical School at University of São Paulo (InCor HC-FMUSP), São Paulo, SP, Brazil.

**Keywords:** Extracorporeal Membrane Oxygenation, Evaluation of Results of Preventive Actions, Heart Defects, Congenital, Health Human Resource Training, Cardiovascular Surgical Procedures

## Abstract

**Introduction:**

Post-cardiotomy myocardial dysfunction requiring mechanical circulatory support occurs in about 0.5% of cases. In our environment, the use of extracorporeal membrane oxygenation has been increasing in recent years.

**Objective:**

To evaluate the impact of investment in professional training and improvement of equipment in the rate of weaning from extracorporeal membrane oxygenation and survival.

**Methods:**

A retrospective study. Fifty-six pediatric and/or congenital heart patients underwent post-cardiotomy extracorporeal membrane oxygenation at our institution between November 1999 and July 2014. We divided this period into two phases: phase I, 36 cases (before the structuring of the extracorporeal membrane oxygenation program) and phase II, 20 cases (after the extracorporeal membrane oxygenation program implementation) with investment in training and equipment). Were considered as primary outcomes: extracorporeal membrane oxygenation weaning and survival to hospital discharge. The results in both phases were compared using Chi-square test. To identify the impact of the different variables we used binary logistic regression analysis.

**Results:**

Groups were comparable. In phase I, 9 patients (25%) were weaned from extracorporeal membrane oxygenation, but only 2 (5.5%) were discharged. In phase II, extracorporeal membrane oxygenation was used in 20 patients, weaning was possible in 17 (85%), with 9 (45%) hospital discharges (*P*<0.01). When the impact of several variables on discharge and weaning of extracorporeal membrane oxygenation was analyzed, we observe that phase II was an independent predictor of better results (*P*<0.001) and need for left cavities drainage was associated with worse survival (*P*=0.045).

**Conclusion:**

The investment in professional training and improvement of equipment significantly increased extracorporeal membrane oxygenation results.

## Abstract (pt)

**Introdução:**

Falência ventricular pós-cardiotomia necessitando de suporte circulatório mecânico ocorre em cerca de 0,5% dos casos. Em nosso meio, o uso de ECMO tem aumentado nos últimos anos.

**Objetivo:**

Avaliar o impacto do investimento na formação profissional e melhoria dos equipamentos na taxa de desmame de ECMO e na sobrevida.

**Métodos:**

Estudo retrospectivo. Cinquenta e seis pacientes cardíacos pediátricos e/ou portadores de cardiopatias congênitas foram submetidos ao implante de ECMO pós-cardiotomia em nossa instituição entre novembro de 1999 e julho de 2014. Nós dividimos este período em duas fases: fase I, 36 casos (antes da estruturação do programa de ECMO) e fase II, 20 casos (após a instalação do programa ECMO com investimento em formação e equipamento). Foram considerados como desfechos primários: o desmame de ECMO e sobrevida até a alta hospitalar. Os resultados em ambas as fases foram comparados pelo teste Chi-quadrado. Para identificar o impacto das diferentes variáveis, ​ foi usada análise de regressão logística binária.

**Resultados:**

Na fase I, 9 pacientes (25%) foram desmamados da ECMO, mas apenas 2 (5,5%) tiveram alta. Na fase II, ECMO foi usado em 20 pacientes, o desmame foi possível em 17 deles (85%), com 9 (45%) altas hospitalares. Quando analisamos o impacto das diversas variáveis ​​sobre a sobrevida e desmame de ECMO, observa-se que a fase II foi um preditor independente de melhores resultados (*P*<0,001) e a necessidade de drenagem das cavidades esquerdas foi associada com pior sobrevida (*P*=0,045).

**Conclusão:**

O investimento na formação profissional e aperfeiçoamento de equipamentos melhorou significativamente os resultados de ECMO em nossa instituição.

**Table:** 

**Abbreviations, acronyms & symbols**	
ABS	Aristotle Basic Score
ACT	Activated clotting time
APTT	Activated Partial Thromboplastin Time
CPB	Cardiopulmonary bypass
ECMO	Extracorporeal membrane oxygenation
ELSO	Extracorporeal Life Support Organization
UFH	Unfractionated heparin

## INTRODUCTION

The first use of extracorporeal membrane oxygenation (ECMO) as respiratory and cardiac support was in 1975^[1]^. Since then this therapy has significantly evolved its indications and results.

However, the high cost of equipment, poor initial results and the need of training specialists have been avoiding ECMO to gain widespread usage in Brazil^[2,3]^.

There are few alternatives for pediatric patients with failure to wean from cardiopulmonary bypass. The intra-aortic balloon pump and prolonged ventricular assist devices have very limited role in children because of sizing characteristics and pediatric patients' physiology, such as high heart rate and elasticity of blood vessels in children^[4]^. That said, ECMO presents as the main alternative for the treatment of refractory post-cardiotomy cardiopulmonary failure^[5]^.

International authors have been reporting satisfactory results with post-cardiotomy ECMO since the late 80's^[6]^. In Brazil, ECMO has been applied consistently in a few centers, but the experience reported in the literature is still scarce^[7]^. According to data from equipment manufacturers, there were commercialized about 200 ECMO membranes in 2013.

The Extracorporeal Life Support Organization (ELSO) guidelines reinforce the importance of using appropriate equipment along with active and continued team training^[8]^.

Patients that demand ECMO are definitely very sick and require a multidisciplinary approach. As it is a relatively new technology, specific staff training before they have contact with these kind of patients is mandatory. But sometimes these steps are skipped, specially in developing countries, due to lack of planning and budget regarding this concern.

In our institution, ECMO has been used since 1999. As above mentioned, we have experienced all sort of drawbacks. However, in 2012, we started restructuring the institutional circulatory assistance program focusing on results improvement. Our initial investment was in team training according to ELSO guidelines and the purchase of specific equipment, especially for the pediatric population.

This study aims to assess the impact of these measures on short-term results of patients undergoing post-cardiotomy ECMO in pediatric patients and patients with congenital heart disease.

## METHODS

Retrospective study including all patients who had undergone post-cardiotomy ECMO [intraoperative or immediate postoperative period (within 24 hours) of pediatric heart surgery or surgery to correct congenital heart disease].

The study was approved by the institutional Ethics Committee (CEP-HC FMUSP 741.911).

Between November 1999 and July 2014, 11,191 patients underwent cardiac surgery to correct congenital heart defects in our institution. In 56 (0.5%) of these patients ECMO was needed in the intraoperative period or within 24 hours after surgery.

### Exclusion Criteria

Patients in whom ECMO was indicated as rescue during cardiac arrest (E-CPR) or at a later period than 24 hours postoperatively were excluded from the analysis.

### Indication

As occurs with most new technologies, its applicability has been modified over time, including improvement of results with accumulation of experience. The indication of ECMO occurred in patients that could not be weaned from the cardiopulmonary bypass (CPB) after clinical support optimization and when the dose of vasopressors and inotropes was progressively higher to maintain vital functions, with persistent metabolic acidosis within 24 hours.

### Contraindication

The presence of uncontrollable bleeding and other ECMO contraindications, hardly ever present in cardiac surgery patients, such as disabling neurological injury or intractable severe extra-cardiac disease.

### Division in two phases

In 2012, there was the implementation of the circulatory assist team (Incor ECMO team), registered in ELSO under the number 276. These changes included investment in equipment and training.

Therefore of that, we divided the patients into two groups: phase I (before the circulatory assist program) with 36 patients and phase II (after implementation of the program) with 20 patients.

The oxygenator used during phase I was silicone membrane (Medtronic inc, Minneapolis, USA) and the pump was Bio-Pump^®^ (Medtronic inc, Minneapolis, USA). Line pressures were not measured at that time and patients under 10 kg did not have a bridge in their circuits.

During phase II the ECMO circuit was updated, the membrane was made of polymethylpentene (Maquet Getting Group, Rasttat, Germany) and the centrifugal pump changed to Rotaflow^®^ (Maquet Getting Group, Rasttat, Germany) with less priming volume and less heat generation. Line pressure measurement was implemented, so was the bridge in circuits for patients under 10 kg.

Staff training in 2012 consisted of two parts. First of all, a group of doctors went to Stollery Children's Hospital and performed a 40 hour ECMO specialist course. After that, these doctors have started training the intensive care nurses following the ECLS specialists' guidelines. From that time on, Canadian specialists started repeating the 40 hour North-American ECMO specialist course inside our hospital once a year with the assistance of our specialists (nurses and doctors).

### Cannulation

Full sternotomy was used and central cannulation performed in all cases. Cannulas were placed in the right atrium and ascending aorta. In cases where the drainage of left chambers was needed, it was chosen to open an interatrial communication or install a second drainage catheter into the left atrium.

The sternum was kept away in all cases and the skin covered by silicon patch or directly approximated using a continuous running suture. The cannulas were exteriorized between skin approximation sutures.

### Anticoagulation protocol

Our protocol consists of an Unfractionated Heparin (UFH) loading dose of 50 to 100 units/kg followed by a 20 to 50 units/kg/hour maintenance dose. UFH dose is adjusted aiming an Activated Clotting Time (ACT) between 180 and 220 seconds, Activated Partial Thromboplastin Time (APTT) between 50 and 80 seconds and an anti-Xa between 0.3 and 0.6 units/mL. ACT is measured every two hours, APTT every 12 hours and anti-Xa daily. When it is not possible to wean the patient from cardiopulmonary bypass, half the dose of protamine is administered and ACT, APTT and anti-Xa levels above mentioned are pursued. If bleeding persists, no more protamine is administered, but other coagulation factors abnormalities are corrected.

### Weaning protocol

During phase I, weaning was based primarily on cardiac function recovery on echocardiography and clinical data (serum lactate, arterial pressure, central venous pressure, urine output). In phase II, measurement of left ventricle outflow tract VTI (Velocity Time Integral) in cm was added to the weaning protocol. This measurement is taken daily with full ECMO flow and 30% of ECMO flow. When it is more than 10 cm, weaning is planned, respecting all abovementioned parameters^[9]^. Hemodynamic stability should be assured for at least 6 hours with low flow. After this period, blood gas samples are collected and the echocardiographic examination is repeated. The patient is decannulated if it meets all the abovementioned criteria. After decannulation we rather not approximate the sternum at this time, in order to avoid compression of the heart cavities and to facilitate possible re-cannulation. Sternum closure is attempted after 24 hours of decannulation and clinical stability. It is important to re-connect the arterial and venous tubes immediately after decannulation and keep the membrane and the pump running for 24 hours, which avoid thrombus formation and allows re-connect the patient to the same circuit in case of clinical deterioration during these critical hours.

### Diagnosis and group comparison

We listed the diseases treated surgically in Table 1. In order to assess if the groups were similar, we compared median age, weight, neonatal rate, gender, Aristotle Basic Score (ABS) and rate of palliative surgery in each group^[10]^.

**Table 1:** List of diseases that led to surgery at each phase.

Diagnosis	Phase I (n= 36)	Phase II (n=20)
TGA	7	1
HLHS	7	1
ToF with PV agenesis	2	2
Truncus Arteriosus	3	1
AVSD	4	2
PA	3	2
Single Ventricle	3	1
Mitral valve disease	3	2
Dilated Cardiomiopathy	1	1
IAA	2	1
AVSD + ToF	0	1
ALCAPA	1	1
ccTGA	0	1
Restrictive Cardiomiopathy	0	1
ASD + VSD + PH	0	1
late post-op ToF PR	0	1

TGA=transposition of the great arteries; HLHS=hypoplastic left heart syndrome; ToF=tetralogy of Fallot; PV=pulmonary valve; AVSD=atrio-ventricular septal defect; IAA=interrupted aortic arch; ALCAPA=anomalous origin of the left coronary artery from the pulmonary artery; ccTGA=congenitally corrected transposition of the great arteries; ASD=atrial septal defect; VSD=ventricular septal defect; PH=pulmonary hypertension; Post-op=postoperative period; PR=pulmonary regurgitation

### Parameters analyzed

ECMO duration was compared between those who were weaned or died. Complications related to ECMO, survival to weaning and discharge home in both phases were analyzed. It was considered as weaning from ECMO when it was possible to perform decannulation and the patient didn't die or returned to ECMO within 24 hours. Timing of cannulation was divided in perioperative cannulation and postoperative cannulation when it occurred within 24 hours after surgery.

### Statistical Analysis

Normality test used was Shapiro-Wilk. Descriptive statistics data are presented as average plus or minus standard deviation for continuous variables with normal distribution and median with interquartile range (IQR) for non-normal distribution and ordinal variables. The comparison between groups including age, neonatal rate, gender, weight, time of cannulation, palliative surgery, left heart drainage and ABS was performed using non-parametric tests (Mann-Whitney and Chi-square). Binary Logistic regression was used to evaluate the impact of phase, age, neonatal rate, gender, weight, time of cannulation, palliative surgery rate, left heart drainage and ABS on weaning from ECMO and survival to hospital discharge results. It was considered significant a *P* value of <0.05. The statistical software used for analysis was SPSS v19.0 (IBM corporation, Armonk-New York; United States).

## RESULTS

Comparison between groups didn't show any statistically significant difference (Table 2). Nevertheless, median ABS in phase I was 10 (9.1-11) and in phase II was 9.3 (7.8-10). This result was borderline statistically different (*P*=0.05), so was the neonatal rate in both phases (38.9% x 15%; *P*=0.06). The surgeries performed in each group are listed in Table 3.

**Table 2:** Demographic and surgical characteristics of patients in phases I and II.

Characteristics	Phase I (n=36)	Phase II (n=20)	*P* value
Age (days) (Median (IQR))	90 (9.5-1637.5)	240 (67.5-2045)	0.17
Weight (kg) (Median (IQR))	4.7 (3.2-17.1)	5.6 (3.2-16.1)	0.57
Neonates (n (%))	14 (38.9%)	3 (15%)	0.06
Male gender (n(%))	22 (61.%)	11 (55%)	0.66
ABS (Median (IQR))	10 (9.1-11)	9.3 (7.8-10)	0.05
Palliatives (n (%))	10 (27.7%)	7 (35%)	0.57
Left drainage (n (%))	4 (11.1%)	4 (20%)	0.36
POI Timing (n(%))	11 (30.5%)	2 (10%)	0.08

IQR=interquartile range; ABS=Aristotle Basic Score; Palliative=palliative surgery; Left Drainage=need for drainage of the left cavities; POI Timing=ECMO implants that did not occur immediately post-bypass but within 24 hours of postoperative period

**Table 3:** Details of surgical procedures performed in two phases.

Surgery	Phase I (n= 36)	Phase II (n=20)
ASO	7	0
Norwood procedure	7	1
ToF + PV agenesis correction	2	2
Truncus Arteriosus correction	3	1
AVSD correction	4	2
Rastelli procedure	3	1
Damus-Kaye-Stensel procedure	1	1
MV replacement	3	3
Cardiac Transplant	1	2
IAA correction	2	1
AVSD + ToF correction	0	1
ALCAPA correction + MV repair	1	1
ASD + PV repair	0	1
PV replacement	0	1
Senning operation	0	1
Extracardiac Fontan procedure	1	0
Glenn procedure	1	0
Rastelli + unifocalization	0	1

ASO=arterial switch operation; ToF=tetralogy of Fallot; PV=pulmonary valve; AVSD=atrioventricular septal defect; MV=mitral valve; IAA=interrupted Aortic Arch; ALCAPA=anomalous left coronary artery from pulmonary artery; ASD=atrial septal defect

The total time on ECMO in patients in whom weaning was possible didn't differ between groups. Average time in phase I was 110.7±52.9 hours, while in the 17 weaned patients in stage II it was 182.2±117 hours (*P*=0.1).

ECMO related complications were similar between groups (Table 4).

**Table 4:** Complications in post-cardiotomy ECMO in both phases.

Complication	Phase I (n=36)	Phase II (n=20)	P value
Bleeding	15 (41.7%)	4 (20%)	0.1
Neurological	3 (8.3%)	3 (15%)	0.4
Renal	3 (8.3%)	2 (10%)	0.8

Bleeding=need for re-thoracotomy and bleeding revision; Neurological =clinically and/or imaging detectable neurological injury; Renal=renal impairment with need for dialysis

**Fig. 1:**
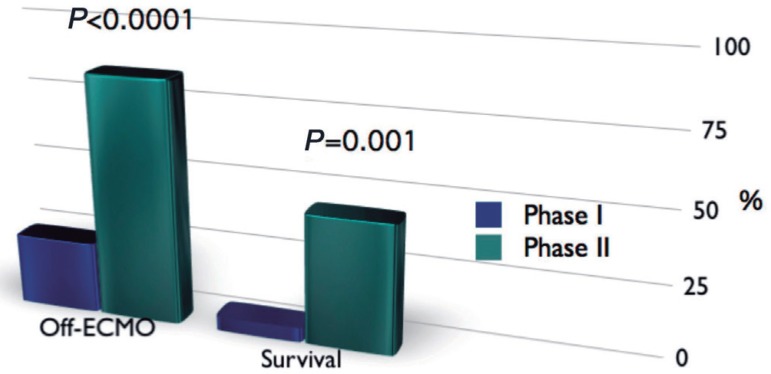
Comparison Chart showing weaning from ECMO and survival results in phases I and II.

In phase I, nine patients (25%) were weaned from ECMO, however, discharge from hospital occurred in only 2 (5.5%). Moreover, in the last 20 patients (phase II), it was possible to wean 17 patients from ECMO (85%, *P*<0.0001) and 45% (9 patients) was discharged from hospital (*P*=0.001) (Figure 1).

P<0.0001 in the Chi-Square test comparing Phase I and II for Off-ECMO results; P=0.001 in the Chi-Square test comparing Phase I and II for survival to hospital discharge.

Binary logistic regression revealed that the need for left cavities drainage didn't impact ECMO weaning, but accounted for an increase in mortality (*P*=0.045, Table 5). Phase II was an independent predictor of ECMO weaning (*P*=0.001) and hospital discharge (*P*=0.01; Table 5).

**Table 5:** Binary Logistic regression analysis of variables in the results.

	Off-ECMO		Survival	
Variables	*P* value	OR (95% CI)	*P* value	OR (95% CI)
Phase II	0.001	26.8 (3.8-185.3)	0.014	108.7 (2.5-4657.7)
Age	0.22	1 (1-1.01)	0.33	0,99 (0.99-1,0)
Weight	0.28	0.9 (0.79-1.07)	0.20	1.2 (0.92-1.54)
Neonate	0.19	3.9 (0.49-32.0)	0.56	2.9 (0.74-120.5)
Gender (M)	0.77	0.78 (0.15-4.07)	0.14	0.19 (0.21-1.77)
Palliative	0.21	4.06 (0.44-37.23)	0.38	0.31 (0.02 - 4.34)
Timing	0.78	0.79 (0.14-4.59)	0.14	22.9 (0.35-1523.7)
Left Drainage	0.88	0.84 (0.09-7.45)	0.045	0.08 (0.007-0.94)
ABS	0.86	0.96 (0.64-1.46)	0.81	1.09 (0.54-2.24)

ABS=Aristotle Basic Score; Off-ECMO=patients weaned from ECMO that were alive and off-ECMO after 24 hours; OR=odds ratio; CI=confidence interval; Neonate=percentage of neonate patients in each sample; Gender (M)=percentage of male patients in each sample; Palliative=percentage of palliative surgeries in each sample; Timing=percentage of ECMO implants that did not occur immediatelly postbypass (perioperative) but within 24 hours of postoperative period; Left Drainage=need for drainage of the left cavities

## DISCUSSION

The use of ECMO as a bridge to post-cardiotomy recovery in children is an increasingly comprehensive reality. Large international centers report encouraging results^[11]^, on the other hand, in our scenario, this has not always been replicated^[7]^.

The high cost of technological development and the training of intensive care teams seem to be the greatest obstacles to this progress. Recently, the Toronto Sick Kids group reported their experience with ECMO emphasizing the importance of technological developments and team structure to improve performance^[6]^. The same group have had published some disappointing results almost ten years earlier^[12]^ with a high incidence of neurological impairment and poor survival, which have led them to work on team performance and on improving technology. These investments were eventually paid off as they showed in their recent publication^[6]^.

We clearly corroborate these findings in the present study, by demonstrating that investment in team training combined with a cost-effective investment in technology can bring significant benefits.

In our service, despite our experience initiated in the late 90's, very few members of the multidisciplinary team have demonstrated knowledge of materials and resources, particularly the direct patient caregivers. This scenario has completely changed with the continued training of these professionals and the subsequent improvement of the results. Recent studies using simulation in ECMO training have showed that this increases the confidence of professionals and increases the ability to solve problems^[1,13]^.

### Re-structuring of the Pediatric Circulatory Assist Program

In early 2012, the institution made significant investment in infrastructure for circulatory assistance, mainly in team training, development of new protocols and purchase of technological devices.

### Team Training

In mid-2012, a group of four doctors from our institution took part on an ECMO training program in North America, more specifically in Edmonton, Canada, according to the ELSO training standards. From that time on, a task force was created to disseminate the knowledge acquired to everyone involved in ECMO assistance.

In April 2013, six professionals from the US and Canada came to Brazil for the first time and replicated the North American ECMO specialist training course. Nine professionals from our institution were trained on this occasion, including five medical doctors, three nurses and one physiotherapist.

Since then, continuing educational program was created to train all the nursing team focused on ECMO patient care. Several training and re-training programs were offered among their peers. A total of 24 institutional nurses were trained by ELSO guidelines and became able to assist patients on ECMO.

Cognitive and practical tests were applied after each training session. In addition, we have performed simulated scenarios focused on training all multidisciplinary team in order to emphasize the importance of teamwork, so necessary in the care of these patients.

### Specific equipment

Disposable: The disposable material including centrifugal pump, an oxygenation membrane and line circuit with the extensions has evolved considerably over the years. Considering the pediatric circuit, there was the addition of the communication bridge between the arterial and venous lines, which provides a better malleable flow, minimizing the risk of thrombosis in the circuit and allows a more gradual and safe weaning. There was also significant progress of oxygenators, which now have a smaller priming, tolerate higher pressures, have greater durability and ability to filter possible air embolism or clots. The most modern centrifugal pumps will operate a smaller priming, provide less heat and therefore less damage to the blood elements.

"Sprinter-cart": The purchase of two minimized supporting devices called sprinter carts, which accommodates all ECMO equipment with specific location for every device needed for ECMO (centrifugal pump console, centrifugal pump head, hand crank, heat exchanger, membrane oxygenator, pressure monitors, flow meters and heparin infusion pump) allowing ECMO to occupy the smallest possible space on the bedside and facilitate patient transport to exams and interventions.

Pressure Monitors: specific pressure monitors of the ECMO circuit (negative venous pressure, pre-membrane pressure and post-membrane pressure) that were not monitored before, were incorporated in the second phase.

Post-bridge arterial flowmeter: flowmeter suitable for pediatric tubing (¼ inch), which monitors the flow in the arterial line after the bridge. Measuring the flow that actually goes to the patient, and was incorporated into pediatric circuit in phase II.

Our results in Phase I were not satisfactory: we attributed this to staff long learning curve, use of non-ideal equipment and no optimization of human resources. Fairly common obstacles in our environment^[3,4,7]^.

However, with planning and structuring of ECMO care, represented herein by phase II, it was possible to obtain results similar to those reported in the literature^[6,11]^. We aim to further enhance these results, especially regarding hospital discharge. Some authors have reported survival over than 60% in this population^[11]^, however, a survival rate of 50% is accepted as satisfactory in the literature^[6,8]^.

Although we have not identified a higher incidence of thoracotomy for bleeding in the first phase, we realized when evaluating the medical records, that bleeding represented a significant problem in phase I, since it was responsible for many early ECMO discontinuations. Currently, better membranes and pumps, leading to less consumption of coagulation factors and more accurate care, account for better hemostasis management and overall results^[14,15]^. However, bleeding persists as the most common complication in most services as in ELSO recordings^[6,8]^.

We didn't notice any significant difference in time of circulatory support between phases in patients weaned from ECMO. There was a tendency of shorter runs in phase II and it was probably caused by a more structured weaning protocol.

Three patients could not be weaned from ECMO in Phase II, one of which was assisted for 28 days and the device was turned off for non-recovery of ventricular function and occurrence of multiple organ failure, preventing the transplant. In the other two cases, both low weight, one was an eight month-old, 3 Kg cardiomyopathy baby with high immune panel, who underwent heart transplantation and had hyperacute humoral rejection. This patient handling was less than 48 hours since the left ventricle contractility was extremely poor, and even with anticoagulation and draining the left atrium, it was observed recurrent intraventricular thrombus formation, so ECMO was turned off. The other was a neonate who underwent correction of interrupted aortic arch and had a small aortic annulus and presented low cardiac output syndrome. This baby suffered massive cerebral hemorrhage after 4 days assisted and ECMO was discontinued due to reserved prognosis.

It is known that neurological injuries are a relatively common complication in these patients and was directly responsible for death in four of our patients^[12]^. Two of the survivors in this series had significant neurological deficits, however, a satisfactory quality of life is observed in both of them, as for the other survivors. Recent studies have shown that quality of life of ECMO survivors is similar to other congenital heart patients^[16]^.

Among the 17 patients weaned in phase II, 15 have recovered ventricular function while two showed no ventricular recovery after 72 hours of ECMO and were listed and transplanted. Both showed good evolution and could be discharged home.

The most frequent complication in both phases was bleeding requiring a thoracotomy for hemostasis revision in 15 patients (41.7%) in phase I and 4 patients in phase II (20%) (*P*=0.14). The occurrence of detectable neurological complications occurred in three patients in Phase I (8.3%). In two of them ECMO was discontinued, the other was decannulated, but came to death four days later. In phase II, three patients had neurological lesions (15%; *P*=0.6). In one of them ECMO was turned off by major cerebral hemorrhage with brain death. The other two were discharged home with hemiparesis. Both are being followed with partial remission of the deficit.

Regarding anticoagulation protocol, the main difference between phase I and II was the daily measurement of anti-Xa, that has a better correlation to heparin levels than ACT and APTT^[17]^.

Renal impairment and need for dialysis during phase I occurred in three patients (8.3%), while stage II dialysis was used in two phase II patients (10%; *P*=0.9).

The drainage of the left chambers was performed in 4 patients in stage I (11%), in three it was performed surgically opening an atrial septal defect and a ventricular septal communication associated with atrial septal defect in the other, since this was a pulmonary atresia with hypoplastic pulmonary arteries and right ventricular dysfunction. In phase II, left drainage was achieved with the placement of additional drainage cannula into the left atrium in three patients (15%; *P*=0.7) in which the drainage of the left cavities was mandatory because of very important left ventricular dysfunction. In only one phase II patient, the creation of an atrial septal communication was performed. This was a patient with preoperative diagnosis of large atrial septal defect and pulmonary artery aneurysm who underwent corrective surgery and evolved with refractory pulmonary hypertension crisis. Echocardiography performed on the first postoperative day revealed an apical ventricular septal defect with right-left shunt that was not detected preoperatively. This patient returned to surgery in poor clinical conditions, arterial saturation of 65% and with two resuscitated cardiac arrests. In surgery it was decided to re-open the ASD and install ECMO. Weaning was possible after 5 days with arterial oxygen saturation around 80%, however, the patient died due to infection 16 days after decannulation. The need for left cavities drainage was a predictor of mortality in our patients. This is not surprising because these patients tend to present with worse left ventricular function pre-ECMO.

Repositioning of the cannulas was necessary in two patients. A phase I patient needed right atrial cannula repositioning to improve drainage and one phase II patient was submitted to repositioning of the aortic cannula.

### Study Limitations

This is a retrospective study with all its limitations. We tried to make sure if the groups were comparable and noticed that Phase I cases had a tendency to be more complex (more neonates and higher ABS; *P*=0.06 and *P*=0.05). On the other hand, logistic regression didn't show impact of these variables in the results, corroborating previous studies^[18]^.

## CONCLUSION

The structuring of an ECMO service with suitable equipment for the pediatric population and team training was able to increase the probability of post-cardiotomy ECMO weaning and survival.

**Table:** 

**Authors’ roles & responsibilities**	
LAM	Analysis and/or interpretation of data; final approval of the manuscript; implementation of projects and/or experiments
LFC	Analysis and/or interpretation of data; final approval of the manuscript; study design; implementation of projects and/or experiments
CT	Conception and design; implementation of projects and/or experiments
JGP	Final approval of the manuscript; implementation of projects and/or experiments
VAG	Conduct of operations and/or experiments; manuscript writing or critical review of its content
NM	Analysis and/or interpretation of data; study design
FRBGG	Conception and design; implementation of projects and/or experiments
MBJ	Analysis and/or interpretation of data; final approval of the manuscript; study design; implementation of projects and/or experiments

## References

[1] Chan SY, Figueroa M, Spentzas T, Powell A, Holloway R, Shah S (2013). Prospective assessment of novice learners in a simulation-based
extracorporeal membrane oxygenation (ECMO) education program. Pediatr Cardiol.

[2] Bartlett RH, Gazzaniga AB, Jefferies MR, Huxtable RF, Haiduc NJ, Fong SW (1976). Extracorporeal membrane oxygenation (ECMO) cardiopulmonary
support in infancy. Trans Am Soc Artif Intern Organs.

[3] Dearani JA, Neirotti R, Kohnke EJ, Sinha KK, Cabalka AK, Barnes RD (2010). Improving pediatric cardiac surgical care in developing
countries: matching resources to needs. Semin Thorac Cardiovasc Surg Pediatr Card Surg Annu.

[4] Neirotti R (2004). Paediatric cardiac surgery in less privileged parts of the
world. Cardiol Young.

[5] Paul Collison S, Singh Dagar K (2007). The role of the Intra-aortic balloon pump in supporting children
with acute cardiac failure. Postgrad Med J.

[6] Kotani Y, Honjo O, Davey L, Chetan D, Guerguerian AM, Gruenwald C (2013). Evolution of technology, establishment of program, and clinical
outcomes in pediatric extracorporeal membrane oxygenation: the "sickkids"
experience. Artif Organs.

[7] Atik FA, Castro RS, Succi FM, Barros MR, Afiune C, Succi G de M (2008). Use of centrifugal pump and extracorporeal membrane oxygenation
as cardiopulmonary support in pediatric cardiovascular
surgery. Arq Bras Cardiol.

[8] Extracorporeal Life Support Organization Guidelines.

[9] Aissaoui N, Luyt CE, Leprince P, Trouillet JL, Léger P, Pavie A (2011). Predictors of successful extracorporeal membrane oxygenation
(ECMO) weaning after assistance for refractory cardiogenic
shock. Intensive Care Med.

[10] Lacour-Gayet F, Clarle D, Jacobs J, Gaynor W, Hamilton L, Jacobs M, Aristotle Committee (2004). The Aristotle score for congenital heart surgery. Semin Thorac Cardiovasc Surg Pediatr Card Surg Annu.

[11] Beiras-Fernandez A, Deutsch MA, Kainzinger S, Kaczmarek I, Sodian R, Ueberfuhr P (2011). Extracorporeal membrane oxygenation in 108 patients with low
cardiac output - a single-center experience. Int J Artif Organs.

[12] Chow G, Koirala B, Armstrong D, McCrindle B, Bohn D, Edgell D (2004). Predictors of mortality and neurological morbidity in children
undergoing extracorporeal life support for cardiac disease. Eur J Cardiothorac Surg.

[13] Burkhart HM, Riley JB, Lynch JJ, Suri RM, Greason KL, Joyce LD (2013). Simulation-based postcardiotomy extracorporeal membrane
oxygenation crisis training for thoracic surgery residents. Ann Thorac Surg.

[14] McMullan DM, Emmert JA, Permut LC, Mazor RL, Jeffries HE, Parrish AR (2011). Minimizing bleeding associated with mechanical circulatory
support following pediatric heart surgery. Eur J Cardiothorac Surg.

[15] Hoashi T, Kagisaki K, Yamashita K, Tatsumi E, Nishigaki T, Yoshida K (2013). Early clinical outcomes of new pediatric extracorporeal life
support system (Endumo (2000) in neonates and infants. J Artif Org.

[16] Costello JM, O'Brien M, Wypij D, Shubert J, Salvin JW, Newburger JW (2012). Quality of life of pediatric cardiac patients who previously
required extracorporeal membrane oxygenation. Pediatr Crit Care Med.

[17] Nankervis CA, Preston TJ, Dysart KC, Wilkinson WD, Chicoine LG, Welty SE (2007). Assessing heparin dosing in neonates on venoarterial
extracorporeal membrane oxygenation. ASAIO J.

[18] Polimenakos AC, Wojtyla P, Smith PJ, Rizzo V, Nater M, El Zein CF (2011). Post-cardiotomy extracorporeal cardiopulmonary resuscitation in
neonates with complex single ventricle: analysis of outcomes. Eur J Cardiothorac Surg.

